# Type of residual astigmatism and uncorrected visual acuity in pseudophakic eyes

**DOI:** 10.1038/s41598-022-05311-x

**Published:** 2022-01-24

**Authors:** Yumi Hasegawa, Masato Honbo, Kazunori Miyata, Tetsuro Oshika

**Affiliations:** 1 Department of Ophthalmology, Faculty of Medicine, University of Tsukuba, 1-1-1 Tennoudai, Tsukuba, Ibaraki 305-8575 Japan; 2 Miyata Eye Hospital, Miyazaki, Japan

**Keywords:** Diseases, Eye diseases

## Abstract

It is difficult to assess the pure impact of the type of residual astigmatism (with-the-rule; WTR, against-the-rule; ATR, and oblique astigmatism) on uncorrected distance visual acuity (UDVA) in pseudophakic eyes due to different age distribution of patients between those subgroups. We conducted the current study to investigate the association between astigmatism type and UDVA in eyes after cataract surgery with consideration for various confounding factors such as age. Data were retrospectively collected from 1535 pseudophakic eyes with corrected distance visual acuity (CDVA) of 20/20 or better, and spherical equivalent between − 0.125 D and 0.0 D. They were classified based on the pattern of residual refractive astigmatism into four groups; minimum astigmatism (< 0.5 D), WTR, ATR, and oblique astigmatism groups. The stepwise multivariate regression analysis showed that the magnitude of residual refractive astigmatism (standardized partial regression coefficient β = 0.559, p < 0.001), CDVA (β = 0.381, p < 0.001), minimum astigmatism group (β = − 0.188, p < 0.001), and WTR astigmatism group (β = − 0.058, p < 0.001) were significantly associated with UDVA (r^2^ = 0.795). Variables excluded from the multivariate regression model include age, preoperative corneal astigmatism, axial length, anterior chamber depth, intraocular lens power, and postoperative spherical equivalent. These results indicate that UDVA is significantly better in eyes with minimum and WTR astigmatism than in those with ATR and oblique astigmatism, after adjustment for confounding parameters. In pseudophakic eyes, oblique and ATR astigmatism exerts a greater impact on UDVA than WTR astigmatism does, even after controlling for age.

## Introduction

Residual astigmatism is one of the important causes of suboptimum visual functions and patient’s dissatisfaction after otherwise successful cataract surgery. It is widely recognized that the most effective and feasible way to compensate the pre-existing astigmatism at the time of cataract surgery is the use of a toric intraocular lens (IOL)^1,2^. Despite the noted efficacy of toric IOLs, however, up to 28% or 47% of eyes had more than 0.50 diopter (D) of residual refractive astigmatism and up to 6% or 16% had more than 1.00 D of residual refractive astigmatism^3,4^. Residual astigmatism after toric IOL implantation has also been reported to range from 0.00 to 2.25 D depending on the preoperative astigmatism^5^. Berdahl et al.^6^ reported that mean change in uncorrected distance visual acuity (UDVA) was 0.16 logarithm of the minimum angle of resolution (logMAR) per 1.0 D of astigmatism, which corresponds to approximately a half line of visual acuity change (2 letters) with every quarter diopter of cylinder change.

There is a paucity of data on the association between visual function and the type of residual astigmatism, such as with-the-rule (WTR), against-the-rule (ATR), and oblique astigmatism in pseudophakic eyes. A few studies^7,8^ evaluated the impact of WTR and ATR astigmatism on UDVA in pseudophakic eyes, but the influence of oblique astigmatism was not assessed and different age distribution among groups was not considered. The influence of WTR, ATR, and oblique astigmatism on visual function was studied by inducing astigmatism with cylindrical lenses in normal eyes of healthy volunteers^9–11^, but these experiments completely ignore the different age distribution among the astigmatism types. We conducted the current study to assess the influence of the type of residual astigmatism on UDVA in eyes after cataract surgery and IOL implantation, by considering the effects of confounding factors such as age of subjects.

## Subjects and methods

### Patients

We retrospectively collected the data of patients who had undergone cataract surgery using phacoemulsification and monofocal IOL implantation between January 2015 and November 2019 at Miyata Eye Hospital. The inclusion criteria were postoperative CDVA of 20/20 or better, postoperative spherical equivalent between − 0.125 D and 0.0 D, 20 years of age or older, and IOLs implanted in the intact continuous curvilinear capsulorrhexis. Eyes were excluded from the subjects if they had any history of ocular surgery except for cataract surgery or had concomitant ocular pathology that can influence visual functions. Cataract surgery was performed by four experienced surgeons using standard phacoemulsification techniques through a 2.4 ~ 2.75 mm sclerocorneal incision located at 12 o’clock. Hydrophobic IOLs with open-loop configuration and blue-light filtering function were implanted in the capsular bag, and sutures were not used to close the sclerocorneal incision. The patients’ background and surgical results were not different between surgeons, including age of subjects, IOL selection, and the distribution of type of residual astigmatism.

### Data collection

Data were collected and analysed on age, preoperative corneal astigmatism, anterior chamber depth, axial length, IOL power, postoperative UDVA and CDVA, postoperative manifest spherical equivalent, and type and magnitude of postoperative refractive astigmatism. Based on the pattern of postoperative manifest refractive astigmatism, eyes were categorized into four groups; minimum astigmatism (< 0.5 D), WTR astigmatism, ATR astigmatism, and oblique astigmatism groups. The astigmatism in which the steeper meridian was within ± 30 degrees of the vertical axis was defined as WTR, and that with the steeper meridian of ± 30 degrees of the horizontal axis was defined as ATR. All others were considered to be oblique astigmatism.

The study protocol was reviewed and approved by the Institutional Review Board of Miyata Eye Hospital (CS-337-032) and University of Tsukuba (R01-325). The committee waived the requirement for patient informed consent regarding the use of their medical record data in accordance with the regulations of the Japanese Guidelines for Epidemiologic Study issued by the Japanese Government. Instead, the protocol was posted at the outpatient clinic to notify participants about the research. This study was performed according to the tenets of the Declaration of Helsinki.

### Statistical analysis

Decimal visual acuity was converted to logarithm of the minimum angle of resolution (logMAR) scale for statistical analysis. For assessment of data among three groups or more, the multiple comparison test was employed, i.e. one-way analysis of variance (ANOVA) with Bonferroni correction. Correlation between continuous variables was analyzed with the Pearson’s correlation coefficient. The stepwise multivariate regression analysis was performed to evaluate the influence of various factors on postoperative UDVA (dependent variable). Explanatory variables included age, preoperative corneal astigmatism, axial length, anterior chamber depth, IOL power, postoperative CDVA, postoperative spherical equivalent, and magnitude and type of residual refractive astigmatism (minimum, WTR, ATR, and oblique astigmatism). The type of residual astigmatism was converted to dummy variables, such as (1, 0, 0, 0), (0, 1, 0, 0), (0, 0, 1, 0), and (0, 0, 0, 1). Differences with a P value of less than 0.05 were considered statistically significant. The numerical data are presented as the mean ± standard deviation (SD) unless otherwise noted. Statistical analysis was conducted with SPSS Statistics for Windows software (version 27, IBM Corp., Armonk, NY, USA).

## Results

The study comprised the records from 1535 pseudophakic eyes of 1535 patients. The patient demographics are shown in Table 1. There was a significant difference in mean age among groups (p < 0.001); the patents with ATR astigmatism were the oldest, followed by those with oblique, minimum, and WTR astigmatism. The post-hoc test revealed significant differences in age between the minimum and ATR astigmatism groups (p < 0.001), WTR and ATR astigmatism groups (p < 0.001), and ATR and oblique astigmatism groups (p < 0.001).

**Table 1:** Patient demographics by the type of residual refractive astigmatism.

	Type of residual refractive astigmatism			
	Minimum (< 0.5 D)	WTR	ATR	Oblique
Number of eyes	772	150	548	65
Age (years old)	70.2 ± 9.0 (29 ~ 92)	69.1 ± 10.2 (32 ~ 93)	75.8 ± 7.6 (48 ~ 94)	72.4 ± 9.7 (45 ~ 89)
Axial length (mm)	23.76 ± 1.44 (21.00 ~ 30.89)	23.84 ± 1.52 (20.39 ~ 28.10)	23.52 ± 1.09 (20.99 ~ 28.06)	23.50 ± 1.16 (21.56 ~ 27.66)
Anterior chamber depth (mm)	3.06 ± 0.42 (1.56 ~ 4.70)	3.07 ± 0.38 (2.04 ~ 4.12)	2.98 ± 0.43 (1.78 ~ 4.81)	2.97 ± 0.39 (1.98 ~ 3.92)
Preoperative corneal astigmatism (D)	0.78 ± 0.60 (0.00 ~ 3.25)	1.23 ± 0.71 (0.00 ~ 4.75)	0.95 ± 0.59 (0.00 ~ 3.50)	1.04 ± 0.75 (0.25 ~ 4.25)
IOL power (D)	20.3 ± 4.0 (0.0 ~ 28.0)	19.8 ± 4.3 (6.0 ~ 29.5)	21.1 ± 2.9 (6.0 ~ 27.5)	20.7 ± 3.3 (7.0 ~ 25.5)
Postoperative refractive astigmatism (D)	0.00 ± 0.00 (0.00 ~ 0.00)	1.10 ± 0.44 (0.50 ~ 3.00)	1.18 ± 0.45 (0.50 ~ 3.50)	0.97 ± 0.36 (0.50 ~ 2.00)
Postoperative spherical equivalent (D)	0.00 ± 0.00 (0.00 ~ 0.00)	− 0.023 ± 0.049 (− 0.125 ~ 0.00)	− 0.031 ± 0.054 (− 0.125 ~ 0.00)	− 0.028 ± 0.053 (− 0.125 ~ 0.00)
Postoperative UDVA (logMAR)	− 0.127 ± 0.062 (− 0.176 ~ 0.097)	0.063 ± 0.123 (− 0.176 ~ 0.523)	0.115 ± 0.140 (− 0.176 ~ 0.824)	0.096 ± 0.125 (− 0.176 ~ 0.523)
Postoperative CDVA (logMAR)	− 0.127 ± 0.062 (− 0.176 ~ 0.00)	− 0.110 ± 0.068 (− 0.176 ~ 0.00)	− 0.098 ± 0.070 (− 0.176 ~ 0.00)	− 0.096 ± 0.073 (− 0.176 ~ 0.00)

*D* diopter, *WTR* with-the-rule, *ATR* against-the-rule, *IOL* intraocular lens, *UDVA* uncorrected distance visual acuity, *CDVA* corrected distance visual acuity, *logMAR* logarithm of the minimum angle of resolution, mean ± standard deviation (range).

**Figure 1:**
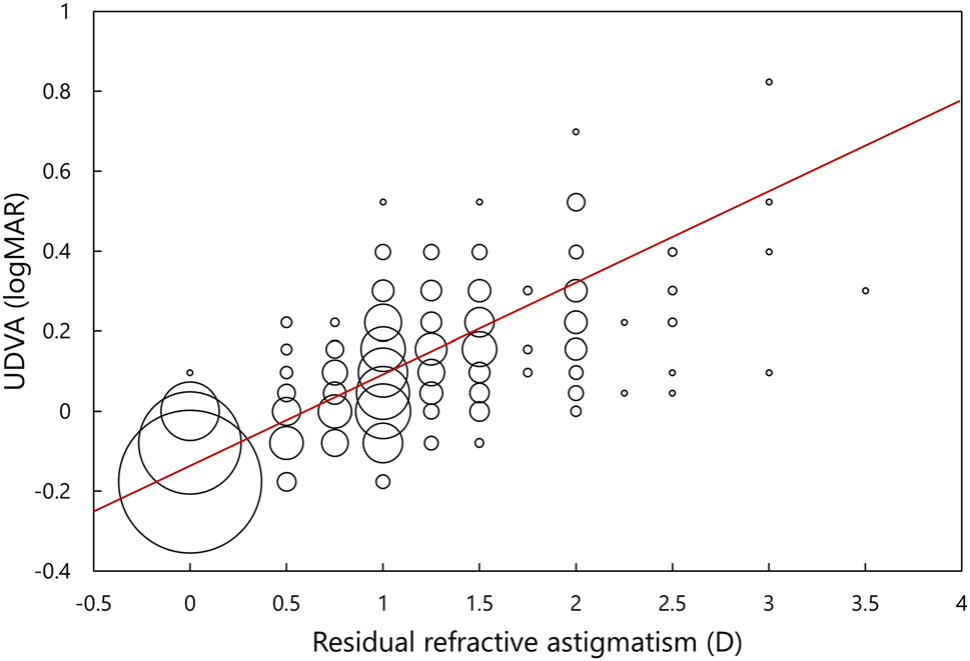
Bubble chart and liner regression of residual refractive astigmatism and UDVA. There was a statistically significant correlation between them (p < 0.001, r^2^ = 0.650). Linear regression analysis provided an equation of UDVA (logMAR) = − 0.122 + 0.193 × residual refractive astigmatism, indicating that the mean change in UDVA with a change of 1.0 D of cylinder was 0.193 logMAR. *UDVA* uncorrected distance visual acuity, *logMAR* logarithm of the minimum angle of resolution.

The bubble chart of postoperative UDVA and the amount of residual refractive astigmatism are shown in Fig. 1. There was a statistically significant correlation between them (p < 0.001, r^2^ = 0.650). Linear regression analysis provided an equation of UDVA (logMAR) = − 0.122 + 0.193 × residual refractive astigmatism, indicating that the mean change in UDVA with a change of 1.0 D of cylinder was 0.193 logMAR.

The stepwise multivariate regression analysis showed that the magnitude of residual refractive astigmatism (standardized partial regression coefficient β = 0.559, p < 0.001), postoperative CDVA (β = 0.381, p < 0.001), minimum astigmatism group (β = − 0.188, p < 0.001), and WTR astigmatism group (β = -0.058, p < 0.001) were significantly associated with postoperative UDVA (r^2^ = 0.795). Variables excluded from the multivariate regression model include age (p = 0.064), preoperative corneal astigmatism (p = 0.966), axial length (p = 0.196), anterior chamber depth (p = 0.279), IOL power (p = 0.174), postoperative spherical equivalent (p = 0.265), ATR astigmatism group (p = 0.667), and oblique astigmatism group (p = 0.667).

## Discussion

In order to elucidate the influence of residual refractive astigmatism on postoperative UDVA, we selected patients with postoperative spherical equivalent between − 0.125 D and 0.0 D and postoperative CDVA of 20/20 or better. If the selection criteria are set to be ± 0.125 D, the anterior and posterior focal lines are located on the opposite side of the retinal plane, and the direction of blurring is reversed on the two foci (vertical versus horizontal), making it difficult to evaluate the pure effects of cylinder axis orientation on vision. Thus, we included only eyes with myopic astigmatism, and excluded those with hyperopic astigmatism.

It was found that UDVA in pseudophakic eyes significantly correlated with the magnitude of residual refractive astigmatism, and their correlation was good (p < 0.001, r^2^ = 0.650). Linear regression analysis noted that UDVA was reduced by 0.193 logMAR for every 1.0 D of cylinder. Berdahl et al.^6^ assessed the effects of residual astigmatism on visual acuity in eyes with toric IOLs, and reported a reduction of 0.16 logMAR per 1.0 D of astigmatism. Similar, but slightly larger values, 0.23 to 0.37 logMAR reduction per 1.0 D of cylinder, have been reported in normal phakic eyes under simulated conditions^12–14^. The current result is in line of these previous findings.

The stepwise multivariate regression analysis showed that postoperative UDVA was significantly associated with the type of residual refractive astigmatism; UDVA was significantly better in eyes with minimum and WTR astigmatism than in those with ATR and oblique astigmatism, after adjustment for age and other factors. Yamamoto et al.^7^ reported that UDVA was worse in pseudophakic eyes with simple myopic ATR astigmatism than in those with simple myopic WTR astigmatism. While UDVA was significantly influenced by the amount of astigmatism in eyes with ATR astigmatism, such association was not found in eyes with simple myopic WTR astigmatism. Until now, the effects of oblique astigmatism on visual function in pseudophakic eyes have not been studied in detail. We herein report that oblique astigmatism in addition to ATR astigmatism is among the factors that can contribute to suboptimum visual function after cataract surgery.

The impact of astigmatism axis orientation on visual functions has been evaluated in healthy phakic eyes by inducing WTR, ATR, and oblique astigmatism with cylindrical lenses. Chen et al.^9^ demonstrated that the reduction in depth discrimination was dependent on the axis of the induced astigmatism, and that the maximum effect occurred with orthogonal-oblique orientations, followed by ATR astigmatism and WTR. Wolffsohn et al.^10^ examined the effect of uncorrected astigmatism in normal adult presbyopes, and reported that eyes with astigmatism at 45- or 180-degree meridian showed worse distance and near visual acuity and subjective-rated clarity than those with astigmatism at 90-degree meridian. In normal volunteers without ocular diseases other than refractive errors, Kobashi et al.^11^ showed that eyes with oblique astigmatism presented significantly worse visual performance than those with WTR or ATR astigmatism. Visual perception is known to critically associated with orientation-specific signals that arise early in the visual processing^15,16^. Humans possess greater behavioral sensitivity to the grating signals with cardinal (horizontal or vertical) orientations than to other orientations, which is called the "oblique effect"^15,16^. Furmanski et al.^16^ assessed asymmetrical responses of human primary visual cortex (V1) to oriented stimuli with functional magnetic resonance imaging, and demonstrated that neural responses in V1 were larger for cardinal stimuli than for oblique (45 and 135 degrees) stimuli. It may be that such effect has played a role in the reduced visual performance with oblique astigmatism than with WTR and ATR astigmatism in the present study. A computer simulation study^11^ created defocused retinal images with astigmatism of different axis orientation using Zemax ray-tracing software (Zemax Development Corp.), and qualitatively demonstrated that it is more difficult to recognize the test letters with oblique orientation than with vertical or horizontal orientations.

The present study has several limitations. First, there are other factors that could influence visual functions of patients but were not included in this study, such as pupil size, irregular astigmatism, monochromatic aberrations, chromatic aberrations, forward and backward scattering, and malpositioning (decentration and tilt) of IOLs. Separate and more extensive studies are needed to clarify the influence of these factors. Second, visual performance other than UDVA was not analysed in this study, such as contrast sensitivity, reading speed, and depth of focus. Third, the distribution of eyes over the type of astigmatism was not even; especially the number of eyes with oblique and WTR astigmatism was considerably smaller than those with ATR and minimum astigmatism. This distribution pattern, however, is similar to those reported in general population^17–20^. Fourth, various types of IOLs were used in this series, and subgroup analyses were not feasible. Since all IOLs were monofocal, non-toric, aspherical, and hydrophobic with open-loop configuration and blue-light filtering function, we believe that differences in IOL type had limited effects on the current results.

In conclusion, we evaluated 1535 pseudophakic eyes with postoperative spherical equivalent between − 0.125 D and 0.0 D and postoperative CDVA of 20/20 or better. There was a statistically significant correlation between postoperative UDVA and the amount of residual refractive astigmatism (p < 0.001, r^2^ = 0.650). The stepwise multivariate regression analysis showed that postoperative UDVA was significantly associated with the type of residual refractive astigmatism; UDVA was significantly better in eyes with minimum and WTR astigmatism than in those with ATR and oblique astigmatism, after controlling for age and other confounding factors.

## Data Availability

The datasets generated during and/or analyzed during the current study are available from the corresponding author on reasonable request.
